# Adjuvant therapy following curative treatments for hepatocellular carcinoma: current dilemmas and prospects

**DOI:** 10.3389/fonc.2023.1098958

**Published:** 2023-04-17

**Authors:** Bin Guo, Qian Chen, Zhicheng Liu, Xiaoping Chen, Peng Zhu

**Affiliations:** ^1^ Hepatic Surgery Center, Tongji Hospital, Tongji Medical College, Huazhong University of Science and Technology, Wuhan, Hubei, China; ^2^ Hepatobiliary Surgery Department, The First Affiliated Hospital of Shihezi University, Shihezi, Xinjiang, China

**Keywords:** hepatocellular carcinoma, curative surgery, tumor recurrence, disease-free survival, adjuvant therapy

## Abstract

Curative surgical treatments, mainly liver resection, are still one of the optimal options for patients with early-, mid-, and even progression-stage hepatocellular carcinoma (HCC). However, the recurrence rate within 5 years after surgery is as high as 70%, especially in patients with high risk factors for recurrence, most of whom experience early recurrence within 2 years. Effective adjuvant therapy may improve prognosis, previous studies found that adjuvant transarterial chemoembolization, antiviral, and traditional Chinese medicine et al. were helpful in preventing HCC recurrence. Nevertheless, due to controversial results or lack of high-level evidence, there is no standardized postoperative management protocol worldwide at present. Continued exploration of effective postoperative adjuvant treatments to improve surgical prognosis is necessary.

## 1 Introduction

Hepatocellular carcinoma (HCC) is one of the most common malignancies worldwide, the number of HCC-related death ranking second among all types of cancers (1, 2). Curative surgery is the ideal treatment protocol for patients with HCC (3, 4), and the choice of surgical indications for liver resection (LR) varies between regions based on different administrative medical evidence. According to the recently updated European guidelines for the treatment of HCC (4), only patients with Barcelona Clinical Liver Cancer (BCLC) stage 0-A HCC are suitable for radical surgery including ablation, LR and liver transplantation (LT), and some patients with stage B HCC are appropriate candidates for LT if they meet extended LT criteria. Strict adherence to BCLC guidelines for surgical indications may prevent many patients from undergoing radical surgical treatment. In contrast, some procedures that exceed BCLC guidelines are often performed in the Asia-Pacific region, including some stage B and stage C patients (5, 6), and some studies have confirmed that surgery has more favorable outcomes than other modalities (7–9). However, a wider range of surgical indications also means a higher probability of accompanying high-risk factors for recurrence.

There are two types of postoperative recurrence, one is early recurrence which is thought to be associated with intrahepatic metastasis from the initial tumor within 2 years after the surgery; the other is late recurrence, which usually occurs after two years due to underlying liver disease like cirrhosis or active hepatitis. Previous studies have shown that portal vein tumor thrombosis(PVTT), microvascular invasion (MVI), and multiple tumors et al. are high-risk factors for early recurrence (10–13), while PVTT and MVI are also high-risk factors for late recurrence (14, 15). Patients with high risk factors are more likely to experience early recurrence, which severely affects the overall outcome of surgical treatment and compromises the patient’s quality of life.

Regarding the management of postoperative adjuvant therapy, there is no standardized strategies worldwide due to unsatisfactory results, controversial findings or lack of high-level evidence (16, 17). Although there are some regimens that may be helpful in reducing postoperative recurrence, such as sorafenib (18, 19), lenvatinib (20), transarterial chemoembolization (TACE) (21), antiviral for hepatitis B-related HCC (22), and Huaier granule (23). However, more well-designed RCTs are required to validate their value. In recent years, the combination of immune checkpoint inhibitors (ICIs) with systemic agents or locoregional therapy has shown excellent anti-tumor effects in the treatment of advanced HCC. This has also led to more options and directions in the study of postoperative adjuvant therapy for HCC, and some relevant studies have recently been preliminarily reported, with overall encouraging results. Here we summarize the current status and recent advances in postoperative adjuvant therapy for HCC.

## 2 The current options and recent advances of adjuvant therapy

### 2.1 Antiviral therapy

#### 2.1.1 Oral antiviral drugs

In Asia, Africa, etc. Hepatitis B virus (HBV) is a major risk factor for the occurrence and progression of HCC. Anti-HBV is an essential basic treatment for HCC, which not only improves liver function and prevents liver fibrosis, but also reduces the recurrence of cured HCC (24). Common oral antiviral drugs include entecavir, tenofovir, adefovir and telbivudine. Huang et al. conducted 2 randomized controlled trials (RCT) confirmed that adjuvant antiviral therapy (telbivudine and adefovir) is helpful in reducing late recurrence for HBV-related HCC after LR, and the OS was also improved (22, 25). Besides, a recent cohort study found that tenofovir maybe a more suitable adjuvant agents for patients received LR than entecavir, which was associated with lower risk of HCC recurrence and better OS (26). For patients with hepatitis C virus (HCV)-associated HCC, the necessity of postoperative adjuvant antiviral therapy remains controversial. A multicenter study which included 47 tertiary care centers in 25 states on the effect of direct-acting antivirals (DAAs) on HCC recurrence did not reach a consistent conclusion (27), 48% responded that DAAs reduce risk, 36% responded that DAAs do not change risk, and 16% responded that DAAs increase risk of HCC recurrence. Similarly, a meta-analysis including 21 studies found no statistically significant difference in the relative risk of DAAs exposure versus no DAAs exposure in preventing HCC recurrence (28). Therefore, postoperative adjuvant antivirals are essential for HBV-related HCC, whereas for HCV-related HCC, there is no high-level evidence to support the necessity of their use.

#### 2.1.2 Interferon

More than a decade ago, many RCTs have explored the value of interferon as an adjuvant therapy due to its antiviral, immunomodulatory, anti-proliferative and anti-angiogenic effects. However, these studies did not obtain consistent conclusions. Two RCTs found adjuvant interferon can’t improve the prognosis for hepatitis viral-related HCC after LR (29, 30); while a RCT found adjuvant interferon improved OS for HBV-related HCC (31), and 2 RCTs found adjuvant interferon improved disease-free survival (DFS) for HCV-related HCC (32, 33). A meta-analysis incorporating only RCTs concluded that adjuvant interferon can improved OS for hepatitis viral-related HCC, but the influence of DFS was modest (34). This may explain why interferon is currently used less frequently. Nevertheless, the immunomodulatory effects of interferon have attracted the interest of researchers. Hu et al. (35) found interferon can release the cytotoxic capacity of T cells by reprogramming glucose metabolism in the HCC tumor microenvironment to enhances the immune response induced by programmed death protein 1 (PD-1) inhibitors. Besides, in a study based on subcutaneous and orthotopic mouse models of HCC, Zhu et al. (36) found a possible synergistic effect of interferon and PD-1inhibitors. The combination of PD-1 inhibitors and interferon is promising both in the treatment of advanced HCC and in adjuvant therapy after radical surgery, and more clinical studies are needed to confirm this conjecture.

### 2.2 Tyrosine kinase inhibitors

Sorafenib is one of the TKIs, which is the first Food and Drug Administrations-approved anti-angiogenic drug for first-line treatment of advanced HCC (3, 37). STORM trial is a large RCT conducted in multiple countries which evaluated the value of adjuvant sorafenib for patients with HCC following LR or ablation, however, the median DFS was 8.5 months in the sorafenib group which was not significantly improved compared with 8.4 months in the placebo group (38). Some subsequent studies have made different findings. Two small sample studies from China found that adjuvant sorafenib following LR significantly improved DFS and OS for patients with BCLC C stage HCC (18, 39). Another propensity score-matched (PSM) study including 718 patients with MVI from China found that adjuvant sorafenib significantly improved DFS and OS compared to LR alone (The 5-years DFS and OS rate was 39% and 57% vs. 19% and 37%) (19). In addition, a recent meta-analysis of 9 studies also concluded that sorafenib is valuable as adjuvant therapy (40). Lenvatinib is another approved TKIs for advanced HCC, which was proved non-inferior to sorafenib, with comparable OS and longer progression-free survival (41). A retrospective study confirmed a similar adjuvant effect of Lenvatinib following LR for patients with MVI (42), which prolong both DFS and OS. Another retrospective study found adjuvant lenvatinib is helpful in improving the prognosis for patients with high residual alpha-fetoprotein following resection or ablation (20). Expanding the indications for surgical treatment in the Chinese region may be an important factor that could explain the inconsistent results with the STROM trial. Patients with high-risk recurrence factors are more deserving of adjuvant therapy and are more likely to benefit from adjuvant TKIs. Of course, this would need to be confirmed by more regional, well-designed RCTs.

### 2.3 Traditional Chinese medicine

Mild medicinal properties and well-tolerance is the major advantage of TCM. TCM has a history of thousands of years, but research on the treatment of HCC and adjuvant therapy is scarce. So far, only 2 RCTs have evaluated the adjuvant therapeutic value of Huaier granules and traditional herbal medicine (THM). Huaier granules plays an anti-tumor role by inhibiting cell proliferation, inducing apoptosis and inhibiting tumor angiogenesis (43, 44). A multicenter RCT including 1,044 patients led by our center explored the postoperative adjuvant value of Huaier granules. Patients with BCLC A or B stage HCC who received adjuvant Huaier granules following radical LR had better DFS and OS than those received LR alone (the 96 weeks DFS and OS rates was 62.39% and 95.19% vs. 42.09% and 91.46%), and the extrahepatic recurrence rate was significantly lower in the Huaier granules group (8.6% vs. 13.1%) (23). Wang et al (45). in a cohort study found that adjuvant Huaier granules was also helpful in improving OS for patients with early-stage HCC after thermal ablation compared with no intervention (The median OS was 35 months vs. 31 months). A recent PSM analysis also confirmed that adjuvant Huaier granules after curative resection were helpful in improving prognosis, especially for patients with tumor diameter >3 cm (after PSM, the 5-years DFS was 42.18% vs. 27.14%) (46). Another RCT conducted by Zhai et al. (47, 48) evaluated the effectiveness of adjuvant THM for patients with small HCC, patients’ DFS and OS were significantly prolonged when receiving adjuvant THM compared with TACE (the median DFS was 85.83 months vs. 26 months). Many active ingredients and mechanisms of action of TCM for the treatment of malignant tumors are still unclear, limiting its widespread clinical use, and application in countries other than China. Continued in-depth exploration is necessary; besides, it is also worth investigating whether the combination of TCM with TKIs or ICIs will produce better anti-tumor effects.

### 2.4 Transarterial interventions

#### 2.4.1 TACE

With more than 40 years of development, TACE is a very mature technique and the standard treatment for intermediate stage (BCLC-B) HCC (4). Postoperative angiography allows timely detection of residual lesions and embolization of the blood supply vessels, which determines the value of TACE as an adjunctive therapy. As early as 1994, an RCT evaluated the value of TACE as adjuvant therapy, patients had significantly longer DFS after receiving 1 postoperative TACE compared to no intervention, but no significant difference in OS (49). Five RCTs were subsequently explored in the next 15 years, but the results were controversial. Two studies found that adjuvant TACE significantly improved DFS and OS (50, 51), one study found that TACE only contributed to OS (52), and one study found that TACE had no significant effect on prognosis (53). Research was stalled for nearly a decade due to controversial results, until the last few years, two RCTs have explored TACE adjuvant therapy in depth and have reached consistent conclusions (54). One was conducted by Wei. et al. evaluated the effect of postoperative adjuvant TACE for patients with MVI and tumor diameter ≥ 5cm, patients who received adjuvant TACE had significantly longer DFS and OS (the median DFS and OS was 14.45 months and 44.29months vs. 9.27 months and 22.37 months) (55). The other RCT which including 280 patients conducted by Wang et al. obtained similarly results, adjuvant TACE effectively reduces postoperative recurrence for patients with high-risk factors (tumor diameter ≥5cm with MVI, or multiple tumors) for recurrence, the median DFS was 25.7 months longer than the control group (49.5 months vs. 23.8 months) (21). Based on these results, TACE was included in the recommended postoperative adjuvant regimen in the Chinese liver cancer treatment guidelines (9). A recent meta-analysis involving 40 studies (10 RCTs and 30 non-RCTs) of postoperative adjuvant TACE noted that patients with high-risk factors for recurrence (MVI, multinodular tumors, and tumor diameter ≥5cm) were more likely to benefit from adjuvant TACE, with longer OS and DFS compared with LR alone; conversely, this study found no improvement in OS, and even worse DFS in patients without MVI (56). Studies in recent years have pointed to the possibility that TACE may help reduce early recurrence for patients with high-risk factors for recurrence, and whether those at low risk of recurrence will benefit from adjuvant TACE is debatable.

#### 2.4.2 Hepatic artery infusion chemotherapy

Identification of suspicious lesions by angiography and continuous infusion of chemotherapy is the modus operandi of HAIC. Izumi et al. (49) first evaluated the effect of HAIC as an adjuvant therapy for patients with MVI and/or intrahepatic metastases after LR in an RCT conducted in 1994, and found that HAIC can effectively prevent postoperative recurrence. A small sample RCT from China obtained similar results in 2015, patients who received adjuvant HAIC had significantly better OS and DFS than surgery alone (57). However, two recently published RCT studies did yield inconsistent results. Li et al. (58) found adjuvant HAIC significantly prolonged the DFS and OS compared without any adjuvant therapy for patients with MVI (the 18 months OS and DFS rate was 97.7% and 58.7% vs. 78.5% and 38.6%). Another RCT found that postoperative adjuvant HAIC having little effect on DFS and OS (59), and this RCT did not specifically select patients with high-risk recurrence factors maybe one reason. Hsiao et al. (60) also found that postoperative adjuvant HAIC did not improve OS and DFS compared with LR alone in a recent retrospective study; while in the subgroup analysis, patients with multiple tumors or MVI were more likely benefited from adjuvant HAIC. A recent meta-analysis confirmed that postoperative adjuvant HAIC is effective in improving prognosis and found that patients with MVI and PVTT are more likely to benefit from it (61). Similar to TACE, the available evidence supports that patients with high-risk relapse factors may be better suited to receive this type of adjuvant therapy.

### 2.5 Radiotherapy

#### 2.5.1 External RT

Narrow pathological margins (< 1 cm), residual tumor tissue/cells or microscopic lesions in the liver that are temporarily undetectable by examination may lead to early recurrence. RT may be helpful in removing these undetectable tumors. In 2014, Yu et al. (62) published the results of the first RCT to assess the effectiveness of postoperative adjuvant RT for patients with narrow margin (< 1 cm), and found that adjuvant RT did not significantly influence the DFS and OS. However, inconsistent conclusions were reached by Gou et al. (63) in a recent retrospective multicenter study that adjuvant RT work a lot in prolonging DFS and OS for patients with narrow or positive surgical margins (the median OS and DFS was 72.5 months and 37.3 months vs. 52.5 months and 24.0 months). With the development of precision RT techniques and the application of new radioisotopes, a variety of external RT modalities have proven to be highly effective for patients with advanced HCC, including intensity modulated RT (IMRT) and stereotactic body RT (SBRT) (64). Recently, Sun et al. (65) found that adjuvant IMRT significantly reduce the postoperative recurrence and prolong the OS for patients with PVTT compared with LR alone (the median DFS and OS was 9.1 months and 18.9 months vs. 4.1 months and 10.8 months). Another single-arm, phase II study also confirmed that postoperative adjuvant IMRT is safe, well tolerated by patients and has a favorable survival prognosis (the 5-year OS and DFS rates were 72.2% and 51.6%) (66). Shi et al. (67) conducted an RCT investigated the prognostic imaging of adjuvant SBRT in patients with MVI who underwent marginal resection, and found that SBRT plays an important role in improving patient’s postoperative prognosis (the 5-years DFS and OS rates in the SBRT group were 56.1% and 75% vs. 26.3% and 53.7% in the control group); besides, most patients were well tolerated with no grade 3 or high adverse effects (AEs) occurred.

#### 2.5.2 Internal RT

Localized implantation with radioactive seeds such as iodine-125 and iodine-131 is a form of internal RT. Between 1999 to 2014, a total of 4 RCTs evaluated the value of iodine-125 or iodine-131 as an adjuvant treatment after radical surgery for HCC, however, these studies did not reach consistent conclusions as well. Two studies concluded that adjuvant iodine-125 and iodine-131 was helpful in improving DFS and OS (68–70), one study concluded that adjuvant iodine-131 was effective in preventing postoperative recurrence but did not prolong OS (71), and one concluded that adjuvant iodine-131 did not influence the survival prognosis (72). Because of the controversial results, iodine-125 and iodine-131 have also been used less frequently in recent years, and relevant studies are lacking. Iodine-131-labelled metuximab is a radiolabeled monoclonal antibody block the CD147 antigen which is associated with a greater susceptibility to metastasis and a worse prognosis for patients with HCC. Li et al. (73) explored its role as an adjuvant therapy for patients with CD147 expression after LR, patients who received adjuvant Iodine-131-labelled metuximab had significantly better DFS and OS compared LR alone (the 5-years DFS and OS rates were 43.4% and 61.3% vs. 21.7% and 35.9%). In the subgroup analysis of this study, patients with high-risk recurrence factors (a solitary tumor of any size with microvascular invasion, satellite nodules, poor differentiation, or two to three nodules) had a significantly better survival prognosis in the adjuvant group than in the control group, whereas no difference in survival prognosis was observed in the intermediate-risk group.

### 2.6 Immunotherapy

Immunotherapies, including lymphocyte infusions, cytokineinduced killer cells (CIK), natural killer cells, tumor vaccines, and ICIs et al. can recognize and kill immune escape tumor cells by regulating or enhancing autoimmune function (74). Immunotherapy has changed the paradigm of human cancer treatment with potent and durable anti-tumor activity in a subset of patients. Many RCTs have evaluated the effectiveness of adjuvant immunotherapy for HCC following LR. In 2000, the first RCT to evaluate immunotherapy as an adjuvant treatment for postoperative HCC released the results, patients who received adjuvant lymphocyte infusions had significant longer DFS compared with placebo (75). Since then, adjuvant immunotherapy was once a hope. A total of 4 RCTs between 2009 and 2016 explored the effectiveness of CIK as an adjuvant treatment after LR, however, these studies did not reach consistent conclusions. Three studies found that adjuvant CIK was helpful in improving postoperative prognosis (76–78); one study, however, discovered that CIK did not prolong postoperative DFS and OS (79).

In recent years, ICIs have made a breakthrough in the treatment of advanced-stage HCC (80, 81). Theoretically, restoring the body’s antitumor cellular immune function is helpful in reducing recurrence after surgery and prolonging the survival time (82). Masatoshi Kudo et al. (83) evaluated the efficacy and safety of adjuvant nivolumab after LR or ablation for HCC in a single-arm study, the median DFS was 26.3 months and the AEs was manageable. And Chen et al. (84) revealed the value of PD-1 inhibitors as adjuvant therapy in a recent cohort study, where patients had significantly better DFS than controls (After PSM, the 2-year DFS was 44.1% vs 21.3%). Similarly, some studies found that adjuvant PD-1 inhibitors also helpful in prolonging DFS for other cancers following surgery such as melanoma, esophageal, and gastroesophageal tumors (85, 86). No relevant RCT study data have been reported on adjuvant PD-1 inhibitors following curative surgery. Besides, the low response rate to PD-1 inhibitors is an important issue to be addressed, and gene sequencing or biomarkers that can predict patient response to PD-1 inhibitors may help identify those patients who are better suited for adjuvant therapy. In addition, there are two issues that should be noted. One is that ICIs may lead to HBV reactivation (87), if ICIs is assisted, regular monitoring for hepatitis B virus and concomitant antiviral treatment is necessary. The other is that ICIs are not commonly used in the perioperative period of LT due to concerns about increased immune rejection. Xie et al. (88) considered that ICIs are not ideal for controlling disease recurrence or *de novo* carcinoma after LT; the immune rejection occurred in 31.9% of patients, with a median OS of only 6.5 months and a mortality rate of 61.7%. In contrast, a meta-analysis of ICIs treatment in solid organ transplant recipients found that approximately 35% of patients faced immune rejection after LT, but this was not the most common cause of death (89). Therefore, adjuvant ICIs after LT should be approached with caution due to the lack of enough evidence.

### 2.7 Combined adjuvant therapy

Postoperative prophylactic TACE or HAIC through angiography can sometimes detect suspicious residual lesions, but it is often powerless for tumor cells shed by intraoperative manipulation and residual tumor cells in the cut edge or blood vessels. This is the reason for carrying out combined adjuvant therapy. Chen et al. (90) explored the feasibility of TACE plus lenvatinib in a multicenter prospective cohort study that screened patients with high risk factors for recurrence to compared the prognosis of receiving combined adjuvant therapy with TACE alone, and the preliminary results are satisfactory (the median DFS was 17.1 months vs. 9 months). There are several well-designed RCTs underway to explore the effectiveness of TACE combined with anti-angiogenic drugs to prevent tumor recurrence (**Table 1**).

**Table 1:** Summary of important ongoing trials on PD-1, and various combination therapies as adjuvant therapy following LR or ablation.

Study ID/Type	Clinicaltrials.gov ID	Eligible patients	Interventions	Primary endpoint
CheckMate9DX (RCT)	NCT03383458	Received LR or ablation and with high-risk factors for recurrence	Nivolumab vs. placebo	DFS
KEYNOTE-937 (RCT)	NCT03867084	Received curative LR or ablation	Pembrolizumab vs. placebo	DFS and OS
JUPITER 04 (RCT)	NCT03859128	Received R0 resection and with high-risk factors for recurrence	Toripalimab vs. placebo	DFS
None (RCT)	NCT05489289	Received LR and with high-risk factors for recurrence	AK104 (anti PD-1/CTLA-4) vs. placebo	DFS
None (RCT)	NCT05240404	Recurrent HCC and received thermal ablation	Toripalimab vs. placebo	DFS
EMERALD-2 (RCT)	NCT03847428	Received curative LR or ablation	Durvalumab + bevacizumab vs. durvalumab + placebo vs. placebo	DFS
IMbrave050 (RCT)	NCT04102098	Received LR or ablation and with high-risk factors for recurrence	Atezolizumab + bevacizumab vs. no intervention	DFS
A-TACE/S-HCC (RCT)	NCT02436902	Received curative LR and with MVI	Sorafenib + TACE vs. no intervention	OS
SOURCE (RCT)	NCT04143191	Received curative LR with resectable advanced HCC	Sorafenib + TACE vs. sorafenib	DFS
DaDaLi (RCT)	NCT04682210	Received curative LR and with high-risk factors for recurrence	Sintilimab + bevacizumab vs. no intervention	DFS
None (RCT)	NCT04639180	Received LR or ablation and with high-risk factors for recurrence	Camrelizumab + apatinib vs. no intervention	DFS
None (RCT)	NCT05367687	Received curative LR or ablation and with high-risk factors for recurrence	Camrelizumab + apatinib vs. camrelizumab	DFS
None (RCT)	NCT05161143	Received LR and with high-risk factors for recurrence	Donafenib + TACE vs. donafenib	DFS
None (RCT)	NCT03839550	Received LR and with high-risk factors for recurrence	Camrelizumab + apatinib vs. HAIC	DFS
None (RCT)	NCT05564338	Received LR and with high-risk factors for recurrence	Sitravatinib + tislelizumab or Placebo + Tislelizumab vs. Placebo	DFS
LANCE (Non-RCT)	NCT03838796	Received LR and with high-risk factors for recurrence	Lenvatinib + TACE vs. TACE	DFS
Y-D202001-0289 (Non-RCT)	NCT05307926	Received curative LR and with high-risk factors for recurrence	Sintilimab + Lenvatinib or sintilimab vs. TACE	DFS
None (Single arm)	NCT04962958	Received LR and with high-risk factors for recurrence	Donafenib + HAIC	DFS
None (Single arm)	NCT05161143	Received LR and with high-risk factors for recurrence	Donafenib + TACE	DFS
ICMJE A (Single arm)	NCT04981665	Received curative LR and with high-risk factors for recurrence	Tislelizumab + TACE	DFS
EMPHASIS (Single arm)	NCT05516628	Received R0 resection and with high-risk factors for recurrence	Atezolizumab + bevacizumab	DFS
CISLD-8 (Single arm)	NCT04418401	Received curative LR and with high-risk factors for recurrence	Donafenib + anti-PD-1 antibody	DFS
ALTER-H006 (Single arm)	NCT05111366	Received R0 resection and with high-risk factors for recurrence	TQB2450(PD L-1) + Anlotinib	DFS
None (Single arm)	NCT05545124	Received R0 resection and with high-risk factors for recurrence	Donafenib + Tislelizumab	DFS
None (Single arm)	NCT05311319	Received curative LR and with high-risk factors for recurrence	Anlotinib + HAIC+ TQB2450	DFS

LR, liver resection; OS, overall survival; DFS, disease-free survival; BCLC, Barcelona Liver Cancer Clinic; PD-1, programmed death protein 1 inhibitors; TACE, transarterial chemoembolization; RCT, randomized controlled trials; HAIC, hepatic artery infusion chemotherapy.

ICIs is the dawn of malignancy treatment in recent years, but has failed successively in phase III trials in advanced HCC (91, 92). Some recent studies found that anti-angiogenic drugs such as TKIs may have synergistic effects with PD-1 inhibitors. TKIs can not only improve antitumor immune responses by modulating macrophages and myeloid-derived suppressor cells to enhance effector T cell responses, but also help to increase the expression of *PD-1* on T cells, thus promoting the action of PD-1 inhibitors (93, 94). Atezolizumab plus bevacizumab (95), Lenvatinib plus Pembrolizumab (81), and Lenvatinib plus Nivolumab (96) et al. have demonstrated stronger anti-tumor effects than single agents in advanced HCC. Xia et al. conducted a single-arm phase 2 study exploring the efficacy and safety of perioperative adjuvant camrelizumab plus apatinib for resectable HCC (97), the 1-year DFS rate of the enrolled patients was 53.85%. In addition, a small RCT including 32 patients conducted buy Zhao et al. (98) published preliminary results in 2021, which confirmed the superiority of camrelizumab plus apatinib compared with HAIC as adjuvant therapy in patients with high-risk recurrence factors following LR (the median DFS was not reached vs. 10.5 months). There are a number of ongoing trials that will more fully evaluate the feasibility of combined adjuvant therapy, such as IMbrave 050 trial, which evaluate atezolizumab plus bevacizumab as adjuvant therapy for high-risk HCC after curative resection or ablation (**Table 1**).

## 3 Discussion

**Figure 1:**
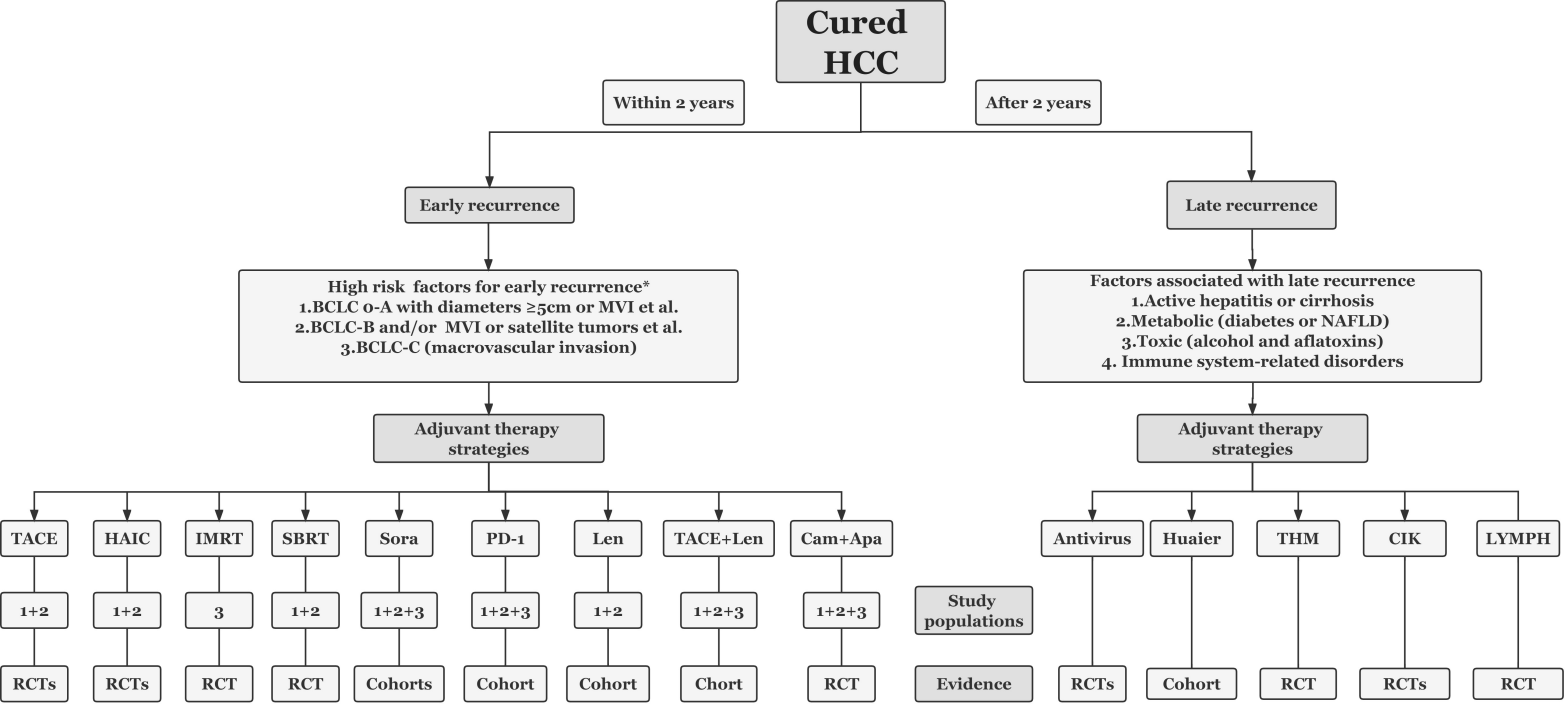
A quick overview of available adjuvant treatment strategies for populations with different risk factors for recurrence. *There are no standardized criteria for high-risk recurrence factors which generally determined by tumor characteristics and highly aggressive pathological features, and highly aggressive pathological features generally refer to poor differentiation, satellite lesions, and microvascular invasion. TACE, transarterial chemoembolization; HAIC, hepatic artery infusion chemotherapy; IMRT, intensity modulated radiotherapy; SBRT, stereotactic body radiotherapy; Sora, sorafenib; PD-1, programmed death protein 1 inhibitors; Len, Lenvatinib; Cam, camrelizumab; Apa, apatinib; NAFLD, non-alcoholic fatty liver disease; THM, traditional herbal medicine; Huaier, huaier granules; CIK, cytokine induced killer cells; LYMPH, lymphocyte infusions.

The high early recurrence rate greatly affects the overall outcome of surgical treatment of HCC, forcing scholars from various countries to continuously explore effective adjuvant treatment strategies. Unfortunately, the currently available regimens are either unsatisfactory in terms of efficacy, controversial in terms of study results, or lack of high-grade evidence. There is still no standard adjuvant treatment protocol worldwide. In reviewing published studies on various adjuvant treatment strategies, it is easy to see that people with high-risk recurrence factors may be better suited for adjuvant therapy, and that combined adjuvant therapy may be more effective than monotherapy. Based on the stratification of risk factors for recurrence, we summarized the improvement of prognosis with current adjuvant treatment options and the sources of evidence (**Table 2**), and a picture was drawn to guide the rapid search for appropriate adjuvant treatment strategies (**Figure 1**). However, two issues are worth noting, one is what exactly is a “high-risk” recurrence factor, and the other is whether the safety of combined adjuvant therapy is acceptable.

**Table 2:** Current adjuvant therapy options, improvement in prognosis, and the level of evidence.

Adjuvant therapy options			Eligible patients	Interventions	Improvement of prognosis	Evidence
**Antiviral therapy**	Oral antiviral drugs	Telbivudine	HBV-related HCC (LR)	Telbivudine vs. no	DFS and OS	RCT (25)
		Adefovir	HBV-related HCC (LR)	Adefovir vs. no	DFS and OS	RCT (22)
		Tenofovir	HBV-related HCC (LR)	Tenofovir vs. entecavir	DFS and OS	R-Cohort (26)
	Interferon	Interferon alpha	HBV-related HCC (LR)	Interferon alpha vs. no	OS	RCT (31)
		Interferon alpha	HCV-related HCC (LR)	Interferon alpha vs. no	DFS	RCTs (32, 33)
**Anti-angiogenic drugs**		Sorafenib	BCLC-C stage (LR)	Sorafenib vs. no	DFS and OS	R-Cohort (18, 39)
		Sorafenib	MVI-positive (LR)	Sorafenib vs. no	DFS and OS	R-Cohort (19)
		Lenvatinib	HBV-related HCC and MVI-positive (LR)	Lenvatinib vs. no	DFS and OS	R-Cohort (42)
		Lenvatinib	High residual alpha-fetoprotein (LR or ablation)	Lenvatinib vs. TACE vs.no	DFS	R-Cohort (20)
**Traditional Chinese medicine**		Huaier granules	BCLC stage A or B (LR)	Huaier granules vs. no	DFS and OS	RCT (23)
		Huaier granules	Early-stage HCC (ablation)	Huaier granules vs. no	DFS	R-Cohort (45)
		Huaier granules	BCLC stage A or B (LR)	Huaier granules vs. no	DFS and OS	R-Cohort (46)
		THM	Solitary HCC <5 cm (LR)	THMvs. TACE	DFS and OS	RCT (48)
**Locoregional therapy**		TACE	MVI and tumor diameter ≥5cm (LR)	TACE vs. no	DFS and OS	RCT (55)
		TACE	Tumor diameter ≥5cm with MVI, or multiple tumors (LR)	TACE vs. no	DFS and OS	RCT (21)
		HAIC	MVI and/or intrahepatic metastases (LR)	HAIC vs. no	DFS	RCT (49)
		HAIC	MVI-positive (LR)	HAIC vs. no	DFS and OS	RCT (58)
**Radiotherapy**	External RT	RT	Narrow or positive surgical margins (LR)	RT vs. no	DFS and OS	R-Cohort (63)
		IMRT	PVTT (LR)	IMRT vs. no	DFS and OS	RCT (65)
		SBRT	MVI-positive (LR)	SBRT vs.no	DFS and OS	RCT (67)
	Internal RT	Iodine-131	Grades I–III according to Okuda staging system (LR)	Iodine-131 vs. no	DFS and OS	RCT (68, 69)
		Iodine-131	Curative surgery (LR or ablation)	Iodine-131 vs. unlabeled lipiodol	DFS	RCT (71)
		Iodine-125	Without vascular/bile duct invasion, tumor nodules ≤ 3(LR)	Iodine-125 vs. no	DFS and OS	RCT (70)
		Iodine-131-labelled metuximab	No macroscopic vascular invasion or extrahepatic distant metastasis and positive CD147 expression (LR)	Iodine-131-labelled metuximab vs.no	DFS and OS	RCT (73)
**Immunotherapy**		Lymphocyte infusions	Stage I, II, IIIA, or IVA according to UICC TNM staging system (LR)	lymphocyte infusions vs. no	DFS	RCT (75)
		CIK	Received curative treatment (LR or ablation or percutaneous ethanol injection)	CIK vs. no	DFS and OS	RCT (76)
		CIK	Resection margin >1 cm, no tumor fracture and hemorrhage, and no tumor distant metastases (LR)	CIK vs. no	DFS	RCT (77)
		CIK	BCLC stage A, B or C (LR)	CIK vs. no	DFS	RCT (78)
		Nivolumab	Received curative treatment (LR or ablation)	Nivolumab	/	Single-arm (83)
		PD-1 inhibitors	MVI, PVTT, satellite nodules, multiple tumors et al. (LR)	PD-1 inhibitors vs. no	DFS and OS	P-Cohort (84)
**Combined adjuvant therap**y		TACE plus Lenvatinib	With macrovascular or bile duct invasion, or MVI alone with multiple tumors et al. (LR)	TACE plus Lenvatinib vs. TACE	DFS and OS	P-Cohort (90)
		Camrelizumab plus apatinib	With PVTT, MVI, or microsatellites (LR)	Camrelizumab plus apatinib vs. HAIC	DFS and OS	RCT (98)

LR, liver resection; OS, overall survival; DFS, disease-free survival; BCLC, Barcelona Liver Cancer Clinic; MVI, microvascular invasion; TACE, transarterial chemoembolization; RCT, randomized controlled trials; HBV, hepatitis B virus; HCV, hepatitis C virus; THM, traditional herbal medicine; HAIC, hepatic artery infusion chemotherapy; RT, radiotherapy; IMRT, intensity modulated radiotherapy; PVTT, portal vein tumor thrombosis; SBRT, stereotactic body radiotherapy; CIK, cytokine induced killer cells; PD-1, programmed death protein 1 inhibitors; R-Cohort, retrospective cohort study; P-Cohort, prospective cohort study.

Although many studies have used “high-risk factors” or “intermediate-risk factors” to select appropriate candidates for adjuvant therapy, there is also a lack of standardized criteria for stratifying the risk of recurrence. The STORM trial incorporated tumor characteristics and pathology reports into the risk level assessment, with high risk recurrence factors including one tumor of any size plus microvascular invasion, satellite tumors, poorly differentiated, or two or three tumors each 3 cm or smaller in size (38). However, many patients with BCLC-stage B or C HCC also underwent radical surgery in some regions, so multiple tumors larger than 3 cm (BCLC-B) and PVTT(BCLC-C) should also been included in high-risk factors. The presence of high-risk recurrence factors means a higher probability of residual tumor in the liver, which is a key factor leading to early recurrence. Effective elimination of residual tumor or prevention of intrahepatic metastasis through postoperative adjuvant therapy will help to reduce early recurrence, which may be one of the reasons why adjuvant therapy is more appropriate for patients with high-risk recurrence factors.

Since the breakthrough of combination therapy in advanced HCC, investigators have not stopped exploring postoperative combined adjuvant therapy. However, the safety of adjuvant therapy cannot be ignored, and whether the remaining liver after LR can withstand the AEs of combined adjuvant therapy also needs to be taken into consideration. In reports of camrelizumab in combination with apatinib for advanced HCC, 77.4% of patients experienced grade ≥ 3 AEs, 28.9% experienced serious AEs, and 2 died (99). In reports of lenvatinib plus pembrolizumab for advanced HCC, 67% of patients experienced grade ≥ 3 AEs, and 3% experienced grade 5 AEs (81). And in the IMbrave 150 study that atezolizumab-bevacizumab caused 56.5% of patients to experience grade 3 or 4 AEs (80). Hypertension and hepatic impairment are the most common AEs. A recent meta-analysis concluded that fatigue, hypertension, and hyperbilirubinemia were more common after combination therapy (100). Triple therapy such as TACE or HAIC combined with TKIs and ICIs has also achieved promising results in the treatment of advanced HCC, but AEs is a more important issue to be aware of. A recent meata analysis of triple therapy suggests that triple therapy is more likely to cause liver function abnormalities and that some potential AEs cannot be evaluated (101). Patients who have undergone surgical trauma have fragile liver function, especially for patients with severe cirrhosis, postoperative adjuvant therapy should not be used blindly as combination therapy or interventional therapy in pursuit of efficacy alone. Tolerability should be assessed more thoroughly when trying new combination regimens, and more regular follow-up is needed. Low response rate is also an urgent issue to be tackled, it makes sense to look for markers that can differentiate the responding population. Previous studies have found that PD-L1 expression, tumor mutation burden and lymphocyte-neutrophil ratio have potential value in differentiating responding populations (102, 103). In addition, extra attention needs to be paid to the fact that drug abuse may cause drug resistance, and resistance to systemic drugs such as sorafenib is now widespread (104).

Overall, the indications for adjuvant therapy should be strictly grasped, and appropriate adjuvant treatment measures should be taken for those with high-risk factors for recurrence. Reasonable stratification of recurrence factors still requires ongoing exploration, and uniform criteria will help in the management of postoperative adjuvant therapy in different regions. Many important ongoing studies are presented in **Table 2**, so keep an eye on the results of these studies and look forward to taking postoperative adjuvant therapy to the next level.

## Author contributions

BG design of the work, drafting the article. QC design charts, critical revision of the article. ZL critical revision of the article. XC critical revision of the article. PZ design of the work, critical revision of the article, final review of the article. All authors contributed to the article and approved the submitted version.
